# Pose Estimation for Visible Light Systems Using a Quadrature Angular Diversity Aperture Receiver

**DOI:** 10.3390/s22145073

**Published:** 2022-07-06

**Authors:** Shengqiang Shen, Jose Miguel Menéndez Sánchez, Shiyin Li, Heidi Steendam

**Affiliations:** 1 School of Information and Control Engineering, China University of Mining and Technology, Xuzhou 221000, China; lishiyin@cumt.edu.cn; 2 Engineering Research Center of Intelligent Control for Underground Space, Ministry of Education, China University of Mining and Technology, Xuzhou 221000, China; 3 Department of Telecommunications and Information Processing, Ghent University/IMEC, Interuniversity Microelectronics Centre, 9000 Ghent, Belgium; jmmenend@espol.edu.ec (J.M.M.S.); heidi.steendam@ugent.be (H.S.); 4 Facultad de Ingeniería en Electricidad y Computación, ESPOL Polytechnic University, Campus Gustavo Galindo Km. 30.5 Vía Perimetral, Guayaquil 90112, Ecuador

**Keywords:** pose estimation, calibration, quadrature angular diversity aperture receiver, Cramér-Rao bound, visible light system

## Abstract

The quadrature angular diversity aperture (QADA) receiver, consisting of a quadrant photodiode (QPD) and an aperture placed above the QPD, has been investigated for pose estimation for visible light systems. Current work on pose estimation for the QADA receiver uses classical camera sensor algorithms well known in computer vision. To this end, however, the light spot center first has to be obtained based on the RSS. However, this is less straightforward than for camera sensors, as in contrast to such sensors where the relationships are linear, the RSS output from the QADA is a non-linear function of the light spot position. When applying closed form solutions or iterative methods for cameras on a QADA, the non-linearity will degrade their performance. Furthermore, since in practice the aperture is not always perfectly aligned with the QPD, a procedure to calibrate the receiver is needed. Current work on calibration requires additional sophisticated equipment to measure the pose during calibration, which increases the difficulty of implementation. In this paper, we target the above problems for pose estimation and calibration of the QADA receiver. To this end, we first study the effect of the strategy of differencing and normalization on the probability density function (PDF), a commonly applied strategy for the QPD’s robustness against RSS variation, and it is shown that the applied strategy results in a complex PDF, which makes an effective and efficient estimation hard to achieve. Therefore, we derive an approximated PDF in a simple closed-form, based on which the calibration and the pose estimation algorithms using the least squares principle are proposed. The proposed calibration does not require any information about the pose of the receiver and is robust to variation of the received power and imperfect knowledge of the radiation pattern of the LED, making it easy to implement. We also derive the corresponding Cramér-Rao lower bound on the misalignment to benchmark the performance of the misalignment and to serve as an indicator to determine the required signal-to-noise ratio (SNR) or number of LEDs to obtain a desired accuracy. The calibration and pose estimation are evaluated by means of a Monte Carlo simulation. Computer simulations show that this theoretical bound is close to the RMSE of the proposed estimator and that the proposed pose estimator outperforms the PnP algorithm.

## 1. Introduction

White LEDs can be modulated up to several MHz. Therefore, they can be used for wireless communication and positioning. As white LEDs are becoming the primary source of light, visible light positioning (VLP) has the potential to achieve positioning with low cost, low power consumption, and long lifetime [1]. Due to the dependency of the RSS values on the pose (position and orientation) of the receiver, it is expected that imperfect knowledge of the pose may strongly affect the performance of visible light communication (VLC) systems [2,3,4,5,6,7]. Recently, a compact receiver based on a quadrant PD (QPD) combined with a single aperture, i.e., the quadrature angular diversity aperture receiver (QADA), was proposed for VLP [8]. The QPD is a segmented photodiode consisting of four matched PDs on a single chip separated by very thin gaps. Combined with an aperture, the resulting receiver achieves angular diversity with a reasonably compact size. Furthermore, as only one aperture needs to be placed above the QPD, the receiver will only have a limited number of construction parameters, making it more robust to construction errors.

The light from an LED reaches the QPD through the aperture only and introduces a light spot of the same size and shape as the aperture. As the position of this light spot is determined by the position of the LED and the pose of the receiver, the overlap area between the light spot and the four quadrants contains information about the pose of the receiver. The RSS values in the four constituent PDs of the QPD are proportional to this overlap area, indicating that a comparison of these RSS values allows us to estimate the angle-of-arrival (AOA) for the different LEDs as well as the position and/or orientation (pose) of the receiver. The current work has been initiated to achieve this goal. Experiments in [8] show that high angular resolution in estimated AOA can be achieved. The positioning performance of the QADA receiver is investigated in several works [8,9,10,11]. The paper [12] considers 3D position estimation with 1D orientation estimation, i.e., it is able to determine the azimuth angle for a receiver that is pointed upwards. The paper [13] that focuses more on the communication aspects considers the effect of a tilt angle, which restricts the orientation to two degrees of freedom. Recently, the work [14] studied the joint 3D position and 3D orientation estimation, namely pose estimation, based on a classic non-iterative perspective-n-point (PnP) algorithm, a well known algorithm for camera sensors in computer vision. In their work, to employ the PnP, first the light spot center is obtained based on the RSS. However, this is less straightforward than for camera sensors, as in such sensors the relationships are linear, whereas the RSS output from the QADA is a non-linear function of the light spot position. Moreover, the strategy of differencing and normalization is commonly applied to the RSS, claimed by most works to improve the robustness against RSS variation, which further increases the non-linearity. These effects will degrade the performance of the PnP algorithm when applied to the QADA receiver; the PnP algorithm is sensitive to noise and as a result it lacks precision [15].

Furthermore, since in practice the aperture is not always perfectly aligned with the QPD, a procedure to calibrate the receiver is needed. The current work starts to consider the presence of misalignment between the aperture and the QPD. In [8], the authors manually adjusted the position of the aperture as a pre-step in the experiment, and the need for calibration is further emphasized in [9]. However, such a hardware calibration is a burdensome, time-consuming procedure. A better approach is to analyze the effect of the aperture misalignment and compensate for it through signal processing. For example, in [12], the authors propose a calibration method to compensate for the mismatch using signal processing. As the authors assume in this calibration phase that the relative distance between the LED and the receiver is known, additional equipment such as Optitrack is required to determine the ground truth on this distance. This limits the usability of the considered method.

In this paper, we target the above problems for pose estimation and calibration of the QADA receiver. On the one hand, we propose a calibration procedure using signal processing that does not require the knowledge of the pose of the receiver; thus no additional hardware is needed to obtain the ground truth on this pose. On the other hand, a pose estimation algorithm is proposed that directly estimates the pose from the (normalized) RSS values, achieving better performance compared to the method that estimates the light spot center and then applies the camera algorithm. To this end, we first investigate the strategy of differencing and normalization to the RSS, i.e., where we only look at the relative differences between the normalized RSS values to obtain an observation that is robust against variation in received power and offsets common to all quadrants [12]. We investigate the distribution of the observation obtained from the noisy RSS values, based on which we then propose the algorithm to estimate the pose of the receiver that is more robust to noise.

The main contributions of this paper are as follows:

1. We first model the RSS vector of the QADA at an arbitrary pose using the perspective projection model. With the help of this model, we derive an explicit expression relating the RSS with the intrinsic parameters, i.e., the aperture height and the misalignment of the aperture, and the extrinsic parameters, i.e., the position and orientation of the receiver.
2. We use the strategy of the normalized differences of the RSS values to make the estimator robust against variations in the transmitted power and radiation pattern. To be able to derive a simple estimation algorithm, we replace the complex true PDF of the resulting observations by an approximated Gaussian PDF based on the first order Taylor series approximation of the observation.
3. Using this approximated PDF, we propose a calibration algorithm based on the least squares (LS) principle. The algorithm jointly estimates the intrinsic and extrinsic parameters from which the intrinsic parameters are extracted. This estimation is performed in an iterative way, where the principle of optimization on manifolds is used. After calibration, a simplified version of the estimation algorithm is used to extract the pose, as now we use the calibrated intrinsic parameters as prior knowledge for the misalignment.
4. To evaluate the optimality of the proposed algorithms, we derive the misspecified Cramér-Rao bound (MCRB) to take into account the effect of the approximated PDF. We compare the MCRB with the Cramér-Rao bound (CRB) for the detected RSS values to quantify the performance loss due to using the normalized differences of the RSS values to make the estimator robust against imperfect knowledge of the transmitted power and radiation pattern. The designed algorithms are verified by Monte Carlo simulations.

The rest of the paper is organized as follows. The channel link model and the expressions for the observation and output vectors are presented in Section 2. The approximated PDF and the calibration system model are provided in this section as well. The calibration and the pose estimation algorithms using the LS principle are proposed in Section 3. Subsequently, the theoretical bound is derived in Section 4. The Monte Carlo numerical comparison is given in Section 5. Finally, some concluding remarks are given in Appendix A.

Notation: Scalars are denoted in italic, e.g., *x*. Lowercase boldface indicates a column vector, e.g., x. Uppercase boldface denotes a matrix or a set, e.g., X, with $I_{N}$ representing an N×N identity matrix, and $0_{N\times M}$ representing an N×M zero matrix. The vector $e_{i}$ is a unit basis vector with its ith element being 1, and the operator $\bar{\cdot }$ converts the Cartesian coordinates x into the homogeneous coordinates $\bar{x}={\left[x^{T},1\right]}^{T}$. Matrix transpose and inverse are indicated by superscript T, and −1, respectively. The Euclidean norm is denoted by ∥·∥, and the expectation operator is denoted by E{·}. $\nabla_{x}=\partial /\partial x$ denotes the Del operator, while $\Delta_{x}^{z}=\nabla_{z}\nabla_{x}^{T}$ denotes the Hessian operator. The rectangular function $\Pi \left(\cdot \right)$ is defined as

(1) $$\Pi \left(x\right)\overset{\Delta }{\;=}\left\{\begin{array}{cc} 1, & \left|x\right|\leq 1.\\ 0, & \left|x\right|>1. \end{array}\right.$$

The set of all real numbers is denoted by R. The group of all rotation matrices, i.e., the special orthogonal group, is denoted by SO(3), and the associated Lie algebra is denoted by so(3). The special Euclidean group is denoted by $SE\left(3\right)$. The operator $\cdot^{\wedge }$ converts a 6×1 vector into a member of $\mathrm{se}\left(3\right)$ – the Lie algebra of SE(3) – which is,

(2) $${\left({ [a^{T},b^{T}]}^{T}\right)}^{\wedge }= [\begin{array}{cc} b_{\times } & a\\ 0^{T} & 0 \end{array}]\in R^{4\times 4},\;a,b\in R^{3\times 1},$$

while the operator $\cdot_{\times }$ converts a 3×1 vector into $\mathrm{so}\left(3\right)$, which is,

(3) $${ [\begin{array}{c} a_{1}\\ a_{2}\\ a_{3} \end{array}]}_{\times }= [\begin{array}{ccc} 0 & -a_{3} & a_{2}\\ a_{3} & 0 & -a_{1}\\ -a_{2} & a_{1} & 0 \end{array}].$$

The operators $\cdot^{\vee }$ and $\cdot_{⊗}$ are, respectively, the inverse operators of $\cdot^{\wedge }$ and $\cdot_{\times }$. Additionally, $\exp\left(\cdot \right)$ and $\log\left(\cdot \right)$ are the matrix exponential and matrix logarithm functions, respectively.

## 2. System Description

In this section, we describe a visible light system that is able to detect signals transmitted by different LEDs from which the pose of the receiver can be determined. To avoid interference between the reference signals for pose estimation, we assume the system adopts multicarrier multiplexing based on (DC-biased) sinusoidal waves [16,17], where a portion of the subcarriers is assigned to the LED anchors to be used as reference signals for pose estimation, while the rest of the subcarriers can be used for communication. Each anchor is assigned a different subcarrier, implying that due to the orthogonality of the subcarriers, the reference signals from the different LEDs can be separated in a simple way. We assume the receiver has knowledge of the used subcarrier and position of each LED, e.g., because this information is contained in the reference signal or is available in a database accessible by the receiver.

The rest of this section is organized as follows. We first model the channel link between a single LED and the QADA and give the expressions for the output vector and observation. We further derive the PDF of the observation. As this PDF is too complex for practical estimation purposes, we also derive a simple closed-form approximation. Finally, we design a system model for calibration and discuss the goals of the pose estimation problem.

### 2.1. Receiver Structure

**Figure 1:**
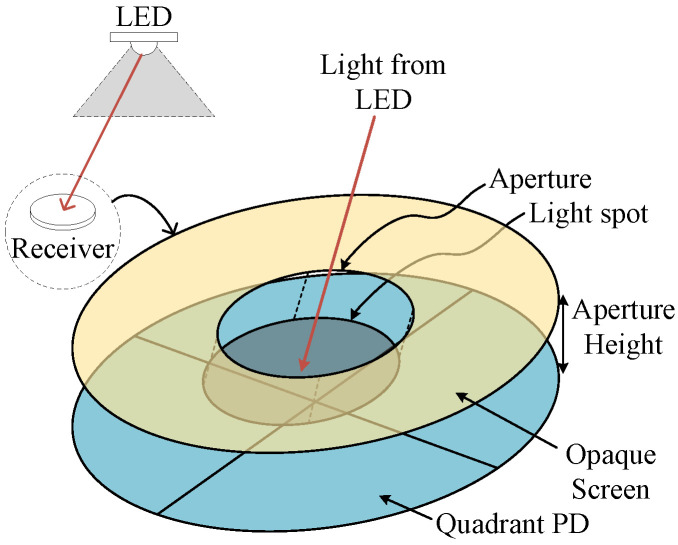
Structure of QADA.

**Figure 2:**
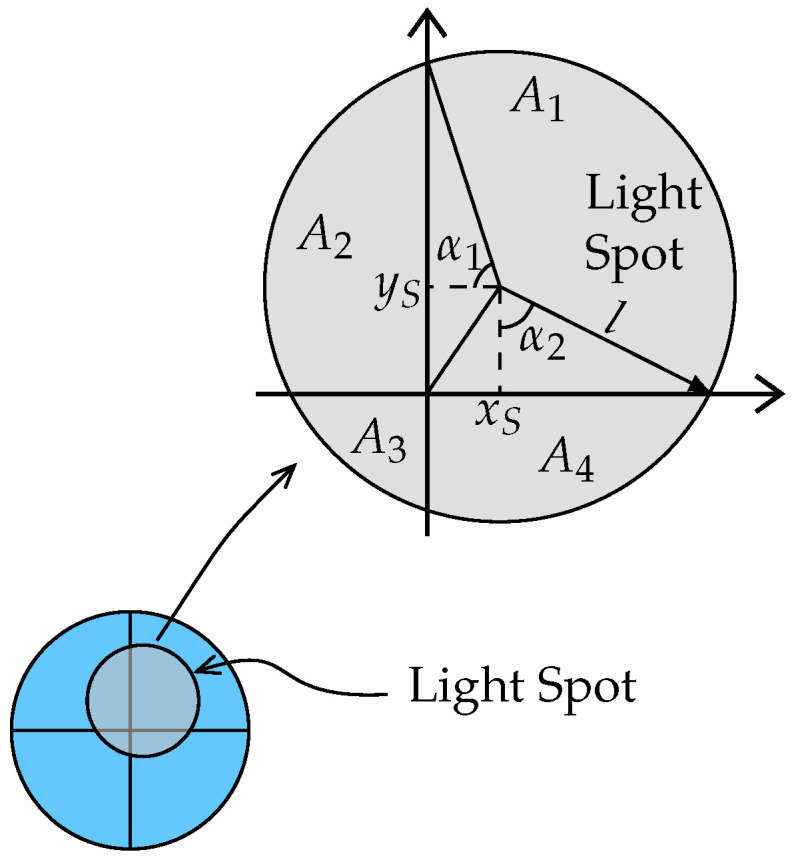
Geometry of the light spot of a light beam, where $x_{S}={\left[r_{S,q}\right]}_{1}$ and $y_{S}={\left[r_{S,q}\right]}_{2}$.

The considered visible light system uses a QPD consisting of four matched photodiodes deposited on a single chip separated by very thin gaps. To convert the incident light of an LED into a light spot on the QPD, we place an opaque screen with a circular aperture at a specific height above and parallel to the surface of the QPD [8] as shown in Figure 1 (a square aperture has the disadvantage of being sensitive to rotational offsets, which means that the rotational offset must be calibrated and compensated for, while a circular aperture is circularly symmetric, implying it is inherently robust to rotational offsets. Therefore, in this paper, we focus on the circular aperture). The light spot on the QPD’s surface, as shown in Figure 2, leads the QPD to produce a unique quad-tuple of RSS values proportional to the overlap areas, i.e., the RSS output is uniquely determined by the position of the light spot. Because of this, the position of the light spot can be estimated from the RSS values, which in turn, e.g., is useful in detecting an object’s angle coordinates [8].

The aperture is chosen so that its diameter is very large compared to the wavelength of the light. Further, as the distance between the LED and the QPD is very large compared to the wavelength, the receiver is in the far-field region of the LED, implying the incident waves can be considered as plane waves. As a result [18], the only light that reaches the QPD is the light that passes through the aperture, and the incident light will introduce a circular light spot of the same size as the aperture on the plane of the QPD. The position of the light spot in the QPD plane not only depends on the position of the light source, but also on the pose of the receiver, implying the RSS outputs in the constituent PDs of the QPD are functions of the position of the light source and the pose of the receiver. We assume that the light spot overlaps with all quadrants and completely falls within the active area of the QPD (in case the light spot does not overlap with all quadrants, one or more of the RSS values will be zero. This will obstruct the strategy of differencing and normalization of the RSS values, which is used to make the estimation algorithm robust against variations in the transmitted optical power. In case the light spot partially falls off the QPD, the relationship between the RSS values and the pose of the receiver becomes very complex, implying the resulting estimation algorithm will have high complexity). To ensure that the first condition is fulfilled, we assume the receiver considers only LEDs that lead all quadrants to produce a sufficiently high RSS output. The second condition implies that the aperture diameter is upper limited by (roughly) the dimension of a quadrant, i.e., half the diameter of a circular QPD or half the side of a square QPD. This guarantees a simple unique relationship between the quad-tuple of RSS values generated by the QPD and the pose of the receiver, and by combining the information from different light sources satisfying the above conditions, the pose of the receiver can easily be determined.

In the following, we will describe the relationship between the pose of the QADA and the position of the light spot, and then we will derive the relationship between the light spot and the RSS output.

### 2.2. Channel Link Model

To obtain the quad-tuple of RSS values as a function of the pose, we note that the dependency of the RSS values on the pose is twofold. Firstly, the channel gain is determined by the radiation pattern of the LED and the irradiance attenuation, implying the incident power at the QPD (and thus the optical power in the light spot) depends on the relative position of the light spot with respect to the LED. Secondly, the RSS values in the quadrants are proportional to the overlap areas of the light spot with the quadrants of the QPD, which is determined by the position of this light spot with respect to the center of the QPD. To express the RSS values as function of the pose of the receiver, we define the following three coordinate systems: (1) The first coordinate system, i.e., the system frame, corresponds to the inertia frame and is used to describe the pose of the receiver with respect to the LEDs. Furthermore, we define two coordinate systems that are attached to the receiver and will be used to describe the position of the light spot and misalignment due to construction errors; (2) In the first receiver attached coordinate system, i.e., the receiver frame, the *x*-*y* plane is the plane of the aperture, and the *z*-axis is the normal to that plane. The origin $O_{r}$ of this receiver frame coincides with the center of the aperture. We will use an extra subscript *r* to indicate coordinates represented in the receiver frame, i.e., the coordinates $x_{r}$ in the receiver frame correspond to their counterpart x in the system frame. This coordinate system is used to find the distance vector of the light spot to the LED as a function of the pose; (3) The second receiver attached coordinate system is the QPD frame, where the *x*-*y* plane is the surface of the QPD, the origin $O_{q}$ is placed in the center of the QPD, and the *x* and *y* axes are aligned with the gaps between the quadrants. We will use an extra subscript *q* to indicate coordinates represented in the QPD frame, i.e., the coordinates $x_{q}$ in the QPD frame correspond to their counterpart x in the system frame. Although this coordinate system is closely related to the receiver frame, its benefit lies in the simplification of the computations, as it enables us to convert the 3D receiver frame coordinates to the 2D coordinates in the QPD frame (we assume that the aperture plane is parallel to the plane of the QPD, and the *x*- and *y*-axes of the QPD and receiver frames are aligned. This implies that the transformation of vectors in the aperture frame into vectors in the QPD frame is a simple translation). This approach is commonly used in the perspective projection model, which we employ to determine the position of the light spot in the QPD plane, and from which we compute the overlap areas of the light spot and the QPD. In the following, we will determine the coordinates of the light spot generated by an LED.

**Figure 3:**
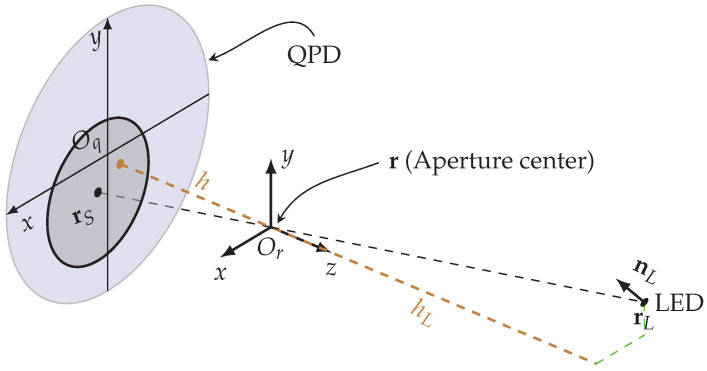
The channel link model. The position of the LED and centers of the aperture and the light spot comply with the perspective projection model, while the shape and size of the light spot is determined by the aperture. Note that in this perspective projection model, the light spot does not correspond to the image of the LED, but it only relates the position of the light spot with the position of the LED.

Assume that in the system frame, the pose of the receiver is described by its orientation R∈SO(3), i.e., R belongs to the Special Orthogonal group and position $r\in R^{3\times 1}$, where the position of the receiver is defined as the position of the center of the aperture. We further assume the LED has position $r_{L}$ and normal vector $n_{L}$ in the system frame. To calculate the position of the light spot in the QPD frame, we notice that the line going through the position $r_{L}$ of the LED and the centers r of the aperture and $r_{S}\in R^{3\times 1}$ of the light spot can be seen as a ray in the perspective projection model of a center of projection located at the center of the aperture and having focal length equal to the aperture height |h|, i.e., the distance between the planes of the aperture and the QPD (see Figure 3). The perspective projection model allows us to relate the 3D coordinates of the LED—the `object’—in the system frame to the 2D coordinates of the light spot —the `image’—in the QPD frame. Note that the position of the light spot in the QPD frame is specified by the (x,y) coordinates only. To make the paper self-contained, we include the background on the perspective projection model. Taking this into account and using the projection principle, the position $r_{S,q}\in R^{2\times 1}$ of the center of the light spot in the QPD frame is given by [19]:

(4) $$r_{S,q}=\overset{C}{\overset{︷}{k_{m,q}+ [\begin{array}{c} e_{1}^{T}\\ e_{2}^{T} \end{array}]\underset{B}{\underset{︸}{ [I_{3},0]\frac{h}{e_{3}^{T}T{\bar{r}}_{L}}\underset{A}{\underset{︸}{T{\bar{r}}_{L}}}}}}}.$$

The matrix $T\in SE\left(3\right)$ is defined through its inverse

(5) $$T^{-1}= [\begin{array}{cc} R & r\\ 0_{1\times 3} & 1 \end{array}].$$

Furthermore, the misalignment vector $k_{m,q}$ expresses the errors in the alignment of the aperture with respect to the QPD. Due to construction errors, a small horizontal offset between the origins of the QPD frame and the receiver frame may occur. This is illustrated in Figure 3, where the brown dot does not coincide with the center $O_{q}$ of the QPD. The horizontal misalignment vector, which takes into account this offset in the transformation of the light spot coordinates in the receiver frame to the coordinates in the QPD frame, is defined as $k_{m,q}≐{\left[u_{m},v_{m}\right]}^{T}$, with $u_{m}$ and $v_{m}$ the misalignment in the *x* and *y* direction, respectively. Taking into account (4), we see that the projection principle consists of three steps:

- Step A: This step converts the LED coordinates ${\bar{r}}_{L}$ in the system frame to the coordinates ${\bar{r}}_{L,r}=T{\bar{r}}_{L}$ in the receiver frame using the matrix T.
- Step B: This scales ${\bar{r}}_{L,r}$ along the projection line with the factor $\frac{h}{h_{L}}$ where $h_{L}=e_{3}^{T}T{\bar{r}}_{L}$ and gets rid of the last component of the homogeneous coordinates through the 3×4 matrix $[I_{3},0]$, consisting of the 3×3 identity matrix extended with a zero column, as this last component is irrelevant for the determination of the position of the light spot. This results in the position $r_{S,r}= [I_{3},0]\frac{h}{e_{3}^{T}T{\bar{r}}_{L}}{\bar{r}}_{L,r}$ of the light spot in the receiver frame.
- Step C: This transforms $r_{S,r}$ into the 2D coordinates $r_{S,q}$ by discarding the *z* coordinate through the mapping of $r_{S,r}$ on the 2×3 matrix $[\begin{array}{c} e_{1}^{T}\\ e_{2}^{T} \end{array}]$ and adds the misalignment vector $k_{m,q}$.

Equation (4) can be rewritten in the following compact form:

(6) $$r_{S,q}=\frac{KT{\bar{r}}_{L}}{e_{3}^{T}T{\bar{r}}_{L}}.$$

The matrix K, which is used in the perspective projection model, is called the intrinsic parameter matrix and describes the internal properties of a receiver, i.e., that are independent of the pose. It only depends on the misalignment vector $k_{m,q}$ and the aperture height *h*:

(7) $$K= [\begin{array}{cccc} h & 0 & u_{m} & 0\\ 0 & h & v_{m} & 0 \end{array}].$$

On the other hand, the matrix T contains the pose information of the receiver and is independent of the internal properties of the receiver. Therefore, it is called the extrinsic parameter matrix. Similarly, taking into account (4), we can write the position $r_{S,r}$ of the center of the light spot in the receiver frame (in homogeneous coordinates) in the following compact form:

(8) $${\bar{r}}_{S,r}=\frac{K_{0}T{\bar{r}}_{L}}{e_{3}^{T}T{\bar{r}}_{L}}\;\mathrm{with}\;K_{0}= [\begin{array}{cc} hI_{3} & 0\\ e_{3}^{T} & 0 \end{array}],$$

and the position ${\bar{r}}_{S}$ of the light spot in the system frame (in homogeneous coordinates) is obtained through:

(9) $${\bar{r}}_{S}=T^{-1}{\bar{r}}_{S,r}.$$

To determine the RSS values in the quadrants of the QPD, we first need to compute the overlap area between the light spot and the quadrants of the QPD. Let us assume the aperture has radius *l*. Taking into account we already assumed that the light spot completely overlaps with the QPD, i.e., no part of the light spot falls outside the QPD, and that all quadrants overlap with the light spot, the overlap areas can easily be computed based on the position $r_{S,q}$ of the center of the light spot in the frame of the QPD. Defining the overlap area $A_{i}>0$ of the *i*th quadrant, i=1,…,4 and the angles $\alpha_{j}\in \left[0,\pi \right]$ with ${\left[r_{S,q}\right]}_{j}=l\cos\alpha_{j}$, j=1,2, and the operator ${\left[r_{S,q}\right]}_{j}$ as the *j*th element of $r_{S,q}$ (see Figure 2), we obtain the overlap areas by solving the following set of equations:

- The light spot has area $\pi l^{2}$, implying $A_{1}+A_{2}+A_{3}+A_{4}=\pi l^{2}$.
- Taking into account that the area of a circular segment with central angle 2α, α∈[0,π] equals $\frac{l^{2}}{2}\left(2\alpha -\sin2\alpha \right)$, we find that $A_{2}+A_{3}=\left(\alpha_{1}-\sin\alpha_{1}\cos\alpha_{1}\right)l^{2}$ and $A_{3}+A_{4}=\left(\alpha_{2}-\sin\alpha_{2}\cos\alpha_{2}\right)l^{2}$.
- The overlap area $A_{1}$ with the first quadrant is a combination of a circle sector with central angle $\frac{3\pi }{2}-\left(\alpha_{1}+\alpha_{2}\right)$ having an area $\left(\frac{3\pi }{2}-\left(\alpha_{1}+\alpha_{2}\right)\right)\frac{l^{2}}{2}$, a rectangle with area ${\left[r_{S,q}\right]}_{1}{\left[r_{S,q}\right]}_{2}=l^{2}\cos\alpha_{1}\cos\alpha_{2}$ and two triangles with areas $\frac{l^{2}}{2}\cos\alpha_{j}\sin\alpha_{j}$, j∈{1,2}.

Note that, depending whether $\cos\alpha_{j}>0$ (if $\alpha_{j}\in \left[0,\frac{\pi }{2}\right]$) or $\cos\alpha_{j}<0$ (if $\alpha_{j}\in \left[\frac{\pi }{2},\pi \right]$), the above contributions will combine positively or negatively, resulting in the wanted overlap area $A_{i}$. Hence, the overlap area vector $A={\left[A_{1},\ldots ,A_{4}\right]}^{T}$ is the solution of $M_{a}A=s$ with

(10) $$M_{a}= [\begin{array}{cccc} 1 & 0 & 0 & 0\\ 0 & 1 & 1 & 0\\ 0 & 0 & 1 & 1\\ 1 & 1 & 1 & 1 \end{array}]\;\mathrm{and}\;s=l^{2} [\begin{array}{c} \frac{3\pi }{4}-\frac{\alpha_{1}+\alpha_{2}}{2}+\frac{\sin2\alpha_{1}+\sin2\alpha_{2}}{4}+\cos\alpha_{1}\cos\alpha_{2}\\ \left(\alpha_{1}-\sin\alpha_{1}\cos\alpha_{1}\right)\\ \left(\alpha_{2}-\sin\alpha_{2}\cos\alpha_{2}\right)\\ \pi \end{array}].$$

Now that we obtained the overlap areas of the light spot with the PDs of the QPD and related them to the pose of the receiver, the RSS values in the different PDs can be determined. To this end, we define the distance vector between the transmitter and the light spot as $v=r_{S}-r_{L}$. With this definition, we can write the distance *v* between the LED and the light spot, and the incidence angle θ, i.e., the angle between the vector −v and the normal nQ=Re3 of the QPD as:

(11) $$v=∥v∥,$$

(12) $$\cos\left(\theta \right)=-\frac{{\left(T^{-1}e_{3}\right)}^{T}\bar{v}}{∥v∥},$$

where the last equation holds due to the equalities ${ [n_{Q}^{T},0]}^{T}=T^{-1}e_{3}$, obtained from (5), and $n_{Q}^{T}v= [n_{Q}^{T},0]\bar{v}$. The channel gain $h_{i}$ for the ith quadrant of the QPD is given by [20]

(13) $$h_{i}=\frac{\Gamma \left(n_{L},v\right)\cos\theta \Pi_{\theta }}{2\pi v^{2}}A_{i},$$

where $\Gamma (n_{L},v)$ is the radiation pattern of the LED pointing $n_{L}$ and is evaluated at direction v, $\Pi_{\theta }=\Pi \left(\phi /\phi_{FOV}\right)$. The factor $\Pi_{\theta }$ in (13) implies that the QPD can detect the light only when the LED is within its FOV, i.e., $0\leq \phi \leq \phi_{FOV}$. Taking into account (11)–(13), the channel gain vector is given by

(14) $$h\left(k,T\right)=-\frac{\Pi_{\theta }\Gamma \left(n_{L},v\right){\left(T^{-1}e_{3}\right)}^{T}\bar{v}}{{2\pi ∥v∥}^{3}}A,$$

and is a function of the intrinsic parameter vector $k≐{\left[u_{m},v_{m},h\right]}^{T}$ and the pose T. From this channel gain, we obtain the RSS output vector $y={\left[y_{1},\ldots ,y_{4}\right]}^{T}$ in the four PDs:

(15) y=g+η,

where $g=R_{p}P_{t}h$, with $R_{p}$ the responsivity of the QPD, and $P_{t}$ the transmitted power. Due to coupling between the quadrants, the noise components are correlated Gaussian distributed random variables $\eta ∼N(0,\sigma_{\eta }^{2}C)$, where C is the correlation matrix with $C_{i,i}=1$ and $C_{i,j}=\rho$ if i≠j, ρ is the correlation between the noise of different quadrants, which can be determined by comparing the noise values in the different quadrants during an experiment and calculate the resulting correlation, and $\sigma_{\eta }^{2}$ is the noise variance.

In the derivation of g in (15), it is assumed that the transmitted optical power is perfectly known, and the LED is a perfect Lambertian radiator. However, directly estimating the intrinsic and/or extrinsic parameters from the RSS output vector will result in inaccurate estimates. To make the estimator more robust to changes in the incident power, the normalized differences between the RSS values along the two axes can be used, as

(16) $$\left\{\begin{array}{c} t_{x}=\frac{\left(y_{1}+y_{4}\right)-\left(y_{2}+y_{3}\right)}{y_{1}+y_{2}+y_{3}+y_{4}}\\ t_{y}=\frac{\left(y_{1}+y_{2}\right)-\left(y_{3}+y_{4}\right)}{y_{1}+y_{2}+y_{3}+y_{4}} \end{array}\right..$$

The approach (16) will make the observations insensitive to variations in the transmitted optical power and radiation pattern provided that the RSS values are noise-free or affected by a common offset so that the normalized differences of (16) are noiseless observations. However, the noise induced by ambient light in visible light systems is non-negligible, and due to the randomness of the shot and thermal noise, the noise present in the different quadrants is in general not equal. The presence of this non-identical random noise will have an impact on the distribution of $t_{x}$ and $t_{y}$. As the estimation of the intrinsic and extrinsic parameters relies on proper knowledge of this distribution, we need to evaluate the effect of the noise on the distribution to see if the observations (16) are still insensitive to variations in the transmitted power when noise is present. Therefore, in the following section, we will analyze the distribution of the normalized differences $t_{x}$ and $t_{y}$.

### 2.3. Approximation to the PDF of the Observation

To find the distribution of the normalized differences, we first rewrite the numerator and denominator of $t_{x}$ and $t_{y}$ in a more compact form by introducing $m≐{ [m_{1},m_{2},m_{3}]}^{T}=M_{y}g$ and $w≐{ [w_{1},w_{2},w_{3}]}^{T}=M_{y}\eta$ with

(17) $$M_{y}= [\begin{array}{cccc} 1 & -1 & -1 & 1\\ 1 & 1 & -1 & -1\\ 1 & 1 & 1 & 1 \end{array}],$$

implying (16) can be rewritten as $t={ [\frac{m_{1}+w_{1}}{m_{3}+w_{3}},\frac{m_{2}+w_{2}}{m_{3}+w_{3}}]}^{T}$. Taking into account that the components of w are linear combinations of Gaussian distributed random variables, w has a multivariate normal distribution, i.e., $w∼N\left(0,\Sigma_{w}\right)$ with

(18) $$\Sigma_{w}=4\sigma_{\eta }^{2} [\begin{array}{ccc} 1-\rho & 0 & 0\\ 0 & 1-\rho & 0\\ 0 & 0 & 1+3\rho \end{array}].$$

Note that the numerator $\left(m_{3}+w_{3}\right)=\sum_{i}y_{i}$ corresponds to the sum of the RSS values in the different quadrants. Hence, $m_{3}$ and $\sigma_{w3}^{2}≐4\left(1+3\rho \right)\sigma_{\eta }^{2}$ are the average and variance of the total received signal strength, which implies we can define $\xi ≐m_{3}^{2}/\sigma_{w3}^{2}$ as the received signal-to-noise ratio (SNR) at the receiver. Furthermore, we define

(19) $$\mu ≐{ [\frac{m_{1}}{m_{3}},\frac{m_{2}}{m_{3}}]}^{T},$$

i.e., the ratio of the averages of the denominator and numerator of t. Obviously, in the absence of noise, the observation equals t=μ. We will show that:

- μ is independent of the channel gain parameters and the transmitted power.
- At high SNR, the distribution of t is given by $t\dot{∼}N(\mu ,\Sigma )$ with
  (20) $$\Sigma =\xi^{-1}\left(\frac{1-\rho }{1+3\rho }I_{2}+\mu \cdot \mu^{T}\right).$$

Let us first take a closer look at the dependency of μ on the channel gain and the transmitted power. Defining h(k,T)=λA, and taking into account that the factor λ is independent of the considered quadrant, it follows that μ is independent of the channel gain parameters and the transmitted power contained in the factor λ, but only depends on the overlap area vector A. Substituting the solution of $M_{a}A=s$ in (19), we find after some straightforward computations that

(21) $${ [\mu ]}_{i}=\left(1-\frac{2\left(\alpha_{i}-\cos\alpha_{i}\sin\alpha_{i}\right)}{\pi }\right).$$

Hence, the vector μ solely depends on the angles $\alpha_{i}$ that are determined by the coordinates of the center of the light spot in the QPD frame. These coordinates, and therefore also μ, only depend on the intrinsic and extrinsic parameters through (6) but not on the channel gain parameters nor the transmitted power.

To obtain the distribution of t at high SNR, we first notice that $t_{x}=\frac{m_{1}+w_{1}}{m_{3}+w_{3}}$ and $t_{y}=\frac{m_{2}+w_{2}}{m_{3}+w_{3}}$ are both a ratio of two independent normal variables with non-zero means, and as they share the same denominator, they are correlated. The distribution of a single ratio of normal variables has been investigated in the literature, e.g., [21,22,23]. In these works, it is shown that a closed-form expression for the PDF in the general case for such a ratio is very complex as its shape differs significantly with its parameters, i.e., in some cases it resembles the Cauchy distribution, in other cases a normal distribution or a bimodal distribution. Several closed-form approximations were discussed in the literature, e.g., the approximate normal distribution. In this paper, we need the distribution of two correlated ratios of normal variables. As this is an even more complicated situation compared to the single ratio case, it is clear that the closed-form expression for the PDF will be even more complex. To simplify the analysis, we first notice that at high SNR, $\frac{w_{i}}{m_{3}}\ll 1$. Expanding t with respect to w using the Taylor series, keeping up to the linear terms in w, we obtain:

(22) $$t\approx \mu +M_{w}w\;\mathrm{with}\;M_{w}= [\begin{array}{ccc} \frac{1}{m_{3}} & 0 & -\frac{m_{1}}{m_{3}^{2}}\\ 0 & \frac{1}{m_{3}} & -\frac{m_{2}}{m_{3}^{2}} \end{array}].$$

This first-order approximation directly leads to the distribution $t\dot{∼}N(\mu ,\Sigma )$ with $\Sigma =M_{w}\Sigma_{w}M_{w}^{T}$. Substituting $M_{w}$ and $\Sigma_{w}$ (18) in Σ, and using the definition of the SNR ξ, it follows that the covariance matrix Σ reduces to (20).

Following the above analysis, at high SNR we can design an estimator based on μ to achieve robustness against variations in the transmitted optical power and radiation pattern to estimate parameters from t.

## 3. Calibration and Pose Estimation

Using the approximate PDF of the normalized differences derived in the Section 2, we can now estimate the pose of the receiver, taking into account the relations (19) and (21). When the intrinsic parameters are known, the estimation of the pose, i.e., the extrinsic parameters, is straightforward. However, in practice, the intrinsic parameters are not prior known and need to be estimated because inaccurate knowledge of the misalignment parameters may result in significant biases in the pose estimates. As these intrinsic parameters can be considered fixed once the receiver is assembled, the intrinsic parameters can be determined once during a calibration process. The standard approach is to determine the intrinsic parameters assuming the pose of the receiver is known. However, this approach requires accurate knowledge of the pose of the receiver. Although it is possible to accurately determine the pose of the receiver, this requires costly equipment, in particular to determine the orientation of the receiver, and a laborious procedure to determine the pose of the receiver before each measurement. Therefore, we propose in this section a method to estimate the intrinsic parameters not requiring the knowledge of the pose, making it simpler and less costly than the standard calibration procedure. Note that our approach differs from the classical algorithm commonly used in camera-based systems, e.g., the non-iterative PnP algorithm and its iterative version [24], as in our method we directly estimate the pose from the normalized differences, while in the classical algorithm used for cameras, first the position of the light spot is determined, after which the pose is extracted. Although the algorithm used for cameras can be adapted to solve the problem at hand, we expect that this algorithm will perform worse than the algorithm proposed in this paper, as compared to the high resolution pixel measurement from the camera sensor, the RSS output from the QADA typically has a lower SNR. Furthermore, because of the non-linear relationship between the center of the light spot and the RSS values, the algorithm will be suboptimal for the considered system [15,25]. As the proposed method directly estimates the pose from the RSS values, it also differs from classical techniques used to determine the position, i.e., where first the AOA or distances to the LEDs are estimated before determining the position using, e.g., trilateration or triangulation.

### 3.1. Calibration Procedure

**Figure 4:**
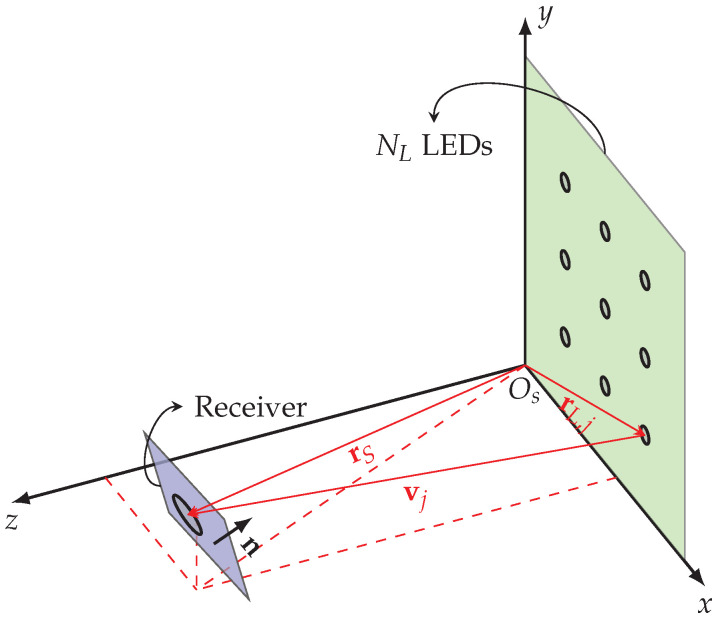
System frame for calibration.

The calibration procedure proposed in this paper makes use of the setup shown in Figure 4, where $N_{L}$ LEDs are installed on a plane in the system frame $xO_{s}y$, and the positions of these LEDs are fixed and known. The receiver observes these LEDs at $N_{T}$ different poses. Hence, the receiver observes for each LED $N_{T}$ quad-tuples of RSS values. The resulting observations are arranged in the $4N_{T}N_{L}\times 1$ vector $\overset{˘}{y}\in R^{4N_{T}N_{L}\times 1}$:

(23) $$\overset{˘}{y}=\overset{˘}{g}+\overset{˘}{\eta },$$

where the vector $\overset{˘}{\eta }$∼$N\left(0,{\overset{˘}{\Sigma }}_{\eta }\right)$ collects the noise, and ${\overset{˘}{\Sigma }}_{\eta }=\sigma_{\eta }^{2}I_{N_{T}N_{L}}⊗C$ (see Section 2.2 for the definition of the noise correlation matrix C). The vector $\overset{˘}{g}$ can be rewritten as $\overset{˘}{g}=R_{p}{ [P_{t,1}g_{1,1}^{T},P_{t,2}g_{1,2}^{T},\ldots ,P_{t,N_{L}}g_{N_{T},N_{L}}^{T}]}^{T}$, where $g_{i,j}\in R^{4\times 1}$ is the channel gain between the jth LED and the receiver at the ith pose $T_{i}^{-1}$, and $P_{t,j}$ is the transmitted power of the jth LED. For each LED and each pose, we compute the normalized differences and arrange them in the $2N_{T}N_{L}\times 1$ vector $\overset{˘}{t}\in R^{2N_{T}N_{L}\times 1}$. Taking into account that the noise in the different observations is statistically independent, we can write the Gaussian approximation of the PDF of $\overset{˘}{t}$ as:

(24) $$p_{m}\left(\overset{˘}{t}\right)=\frac{1}{\sqrt{\det(2\pi \overset{˘}{\Sigma })}}e^{\left(-\frac{1}{2}∥\overset{˘}{t}-\overset{˘}{\mu }{∥}_{\overset{˘}{\Sigma }}^{2}\right)}$$

where $\overset{˘}{\mu }={ [\mu_{1,1}^{T},\mu_{1,2}^{T},\ldots ,\mu_{N_{T},N_{L}}^{T}]}^{T}$. The components $\mu_{i,j}$ correspond to the jth LED and the ith pose and are defined through (19), while the covariance matrix $\overset{˘}{\Sigma }$ is the block diagonal matrix given by

(25) $$\overset{˘}{\Sigma }=\mathrm{diag}\left(\Sigma_{1,1},\Sigma_{1,2},\ldots ,\Sigma_{1,N_{L}},\Sigma_{2,1},\ldots ,\Sigma_{N_{T},N_{L}}\right)$$

In this covariance matrix, $\Sigma_{i,j}$ is the covariance matrix for the noise of $t_{i,j}$ (see (20)). These covariance matrices $\Sigma_{i,j}$ are a function of $\mu_{i,j}$, which in turn depend on the parameters to be estimated. As a consequence, maximum likelihood estimation will be complex. Therefore, we will use the least squares (LS) method to estimate the intrinsic and extrinsic parameters. Let us define the parameter set $\Theta =\{k,T_{1},\ldots ,T_{N_{T}}\}$, then the LS estimate is given by:

(26) $$\begin{array}{ccc} & & \hat{\Theta }=\arg\min_{\Theta }\frac{1}{2}{∥\overset{˘}{t}-\overset{˘}{\mu }∥}^{2},\\ & & s.t.\;R_{i}^{T}R_{i}=I,\;\det\left(R_{i}\right)=+1. \end{array}$$

The constraints $R_{i}^{T}R_{i}=I$ and $\det\left(R_{i}\right)=+1$ imply that $R_{i}$, which defines the orientation of the receiver at pose *i* and is enclosed in the transform matrix $T_{i}^{-1}\in SE\left(3\right)$ (see (5)), is a rotation matrix that is a member of $SO\left(3\right)$. Unfortunately, (26) has no closed-form solution, implying we need to resort to an iterative algorithm to estimate Θ. As the solution of the above (iterative) constrained optimization problem is complex and cumbersome in Euclidean space, we first notice that $SE\left(3\right)$ is a manifold and convert the above optimization problem into an unconstrained optimization problem on manifolds [26]. In what follows, we use the Gauss–Newton method on manifold $SE\left(3\right)$ to solve (26), similarly as in [27]. At each iteration, the update direction is calculated by

(27) $${ [\Delta_{k},\Delta_{T_{1}},\ldots ,\Delta_{T_{N_{T}}}]}^{T}={\left(\nabla_{\Theta }\overset{˘}{\mu }\right)}^{†}\left(\overset{˘}{t}-\overset{˘}{\mu }\right)$$

where ${\left(\cdot \right)}^{†}$ is the Moore–Penrose pseudoinverse, and $\nabla_{\Theta }\overset{˘}{\mu }=[\nabla_{\Theta }\mu_{1,1},\nabla_{\Theta }\mu_{1,2},\ldots ,$$\nabla_{\Theta }\mu_{N_{T},N_{L}}{]}^{T}\in R^{2N_{T}N_{L}\times \left(6N_{T}+3\right)}$ the gradient of $\overset{˘}{\mu }$ with respect to Θ. The derivation of the components $\nabla_{\Theta }\mu_{i,j}$ can be performed in a similar way as in [27], and the result is given in Appendix A. As the update directions (27) depend on k and all $T_{i}$, the intrinsic parameters and the poses need to be estimated jointly:

(28) $$\left\{\begin{array}{c} k^{t+1}=k^{t}+\tau_{k}\Delta_{k}\left(k^{t},T_{1}^{t},\ldots ,T_{N_{T}}^{t}\right)\\ T_{i}^{t+1}=\exp\left({\left(\tau_{T}\Delta_{T_{i}}\left(k^{t},T_{1}^{t},\ldots ,T_{N_{T}}^{t}\right)\right)}^{\wedge }\right)T_{i}^{t},\;i\in \left\{1,\ldots ,N_{T}\right\} \end{array}\right.$$

where $\tau_{k}$ and $\tau_{T}$ control the incremental step size for k and $T_{i}$, respectively.

The Gauss–Newton method needs an initialization $\Theta^{0}$ to start the iterative estimation process. To obtain this initial estimate, we consider the direct linear transformation method [24]. Assuming the positions of at least four LEDs, and the positions of the light spots for these LEDs are known, the direct linear transformation method gives a closed-form coarse estimate for $\Theta^{0}$. Although in our problem, the positions of the light spots are not known, we can estimate them from the normalized differences $t_{i,j}$, which is a noisy version of $\mu_{i,j}$ (22). This $\mu_{i,j}$ is a function of the angles $\alpha_{\ell }$, ℓ=1,2 (21), which in turn are related to the position $r_{S,q,i,j}$ of the light spot, i.e., we can write $\mu_{i,j}$ as a function of $r_{S,q,i,j}$: $\mu_{i,j}\left(r_{S,q,i,j}\right)$. In many situations, the noise is relatively small, implying we can neglect the presence of the noise, i.e., $t_{i,j}\approx \mu_{i,j}$. Inverting the function $\mu_{i,j}\left(r_{S,q,i,j}\right)$, we obtain the coarse estimate ${\hat{r}}_{S,q,i,j}\in R^{2\times 1}$ of the light spot for the jth LED and the ith pose:

(29) $${\hat{r}}_{S,q,i,j}={\left(\mu_{i,j}\right)}^{\left(-1\right)}\left(t_{i,j}\right),$$

where ${\left(\cdot \right)}^{\left(-1\right)}$ is the inversion operator. Unfortunately, due to the non-linearity of μ as a function of its argument $r_{S,q}$, no closed-form expression can be found for this inverse. However, as μ monotonically increases with $r_{S,q}$, we can precompute the function μ for a set of values $r_{S,q}$ and save them in a look-up table. Based on this table, the inverse can be determined by interpolating between the values available in the look-up table.

### 3.2. Theoretical Lower Bound

In the Section 3.1, we considered the LS estimation of the intrinsic parameters based on the approximated PDF of $\overset{˘}{t}$ in the presence of the (unwanted) extrinsic parameters $T_{i}$. To gain insight into the optimality of the designed calibration algorithm and to investigate how the SNR, $N_{L}$ or $N_{T}$ would effect the accuracy of the estimator, in this section we derive the theoretical lower bound for the mean squared errors of the intrinsic parameters *h* and $k_{m,q}$. Since the estimation includes the unwanted parameter $T_{i}$ and since the estimator is designed based on an approximated PDF, we will derive the misspecified Cramér-Rao bound (MCRB) [28] for the whole parameter set Θ and only keep the left upper 3×3 submatrix corresponding to the intrinsic parameters.

The MCRB for the whole parameter set Θ is given by

(30) $$\mathrm{MCRB}\left(\Theta \right)={\left(M_{1}\left(\overset{˚}{\Theta }\right)\right)}^{-1}M_{2}\left(\overset{˚}{\Theta }\right){\left(M_{1}\left(\overset{˚}{\Theta }\right)\right)}^{-1}+\mathrm{Bias}\left(\overset{˚}{\Theta },\Theta \right).$$

In (30), $\overset{˚}{\Theta }$ is the parameter set to which the estimation converges due to the PDF approximation assumed by the estimator, given by

(31) $$\overset{˚}{\Theta }=\arg\min_{\Theta }D\left(p\left(\overset{˘}{t}\right)\left|\right|p_{m}\left(\overset{˘}{t}|\Theta \right)\right),$$

where $D\left(p\left(\overset{˘}{t}\right)\left|\right|p_{m}\left(\overset{˘}{t}|\Theta \right)\right)$ is the Kullback–Leibler divergence (KLD) between the true ($p(\overset{˘}{t})$) and the approximated ($p_{m}\left(\overset{˘}{t}|\Theta \right)$) PDFs. The matrices $M_{1}$, $M_{2}$, and $\mathrm{Bias}(\overset{˚}{\Theta },\Theta )$ are, respectively, given by [28]

(32) $$M_{1}=E_{\overset{˘}{t}}\left\{\Delta_{\Theta }^{\Theta }\ln p_{m}\left(\overset{˘}{t}|\Theta \right),\right\}$$

(33) $$M_{2}=E_{\overset{˘}{t}}\left\{\nabla_{\Theta }^{T}\ln p_{m}\left(\overset{˘}{t}|\Theta \right)\nabla_{\Theta }\ln p_{m}\left(\overset{˘}{t}|\Theta \right),\right\}$$

(34) $$\mathrm{Bias}\left(\overset{˚}{\Theta },\Theta \right)=\epsilon \left(\overset{˚}{\Theta },\Theta \right)\epsilon {\left(\overset{˚}{\Theta },\Theta \right)}^{T},$$

where $\epsilon (\overset{˚}{\Theta },\Theta )$ is the error vector between $\overset{˚}{\Theta }$ and Θ. The error vector is defined as $\epsilon \left(\overset{˚}{\Theta },\Theta \right)={ [\epsilon_{k}^{T},\epsilon_{T_{1}}^{T},\ldots ,\epsilon_{T_{N_{T}}}^{T}]}^{T}$ with $\epsilon_{k}=\overset{˚}{k}-k$ and $\epsilon_{T_{i}}=\log{\left(T_{i}{\overset{˚}{T}}_{i}^{-1}\right)}^{\vee }$. After a few rearrangements, it can be seen that the expectations in (32) and (33) depend on $p(\overset{˘}{t})$ through its mean and covariance only. Due to the complexity of the true PDF $p(\overset{˘}{t})$, this mean and average must be calculated numerically via Monte Carlo integration.

To analyze the effect of $N_{T}$, $N_{L}$ and SNR, we take in account that since the approximated PDF $p_{m}\left(\overset{˘}{t}|\Theta \right)$ is Gaussian, the minimum of the KLD (31) is obtained when the first two moments of the true and the approximated PDFs are matched [29]. When the SNR is high, the first two moments of the approximated PDF approach those of the true PDF. As a result, the estimator is asymptotically unbiased, i.e., $\mathrm{Bias}(\overset{˚}{\Theta },\Theta )\approx 0$ for SNR ≫1, so that $M_{1}\approx M_{2}$, $\mathrm{MCRB}\left(\Theta \right)\approx M_{2}^{-1}$ and

(35) $$M_{2}\approx \sum_{i}^{N_{T}}\sum_{j}^{N_{L}}\nabla_{\Theta }^{T}\mu_{i,j}\Sigma_{i,j}^{-1}\nabla_{\Theta }\mu_{i,j}.$$

It can be seen that when $N_{T}$ or $N_{L}$ increases, the number of terms in (35) increases. As the matrices being summed in (35) are symmetric and positive-semidefinite, this implies that $M_{2}$ is non-decreasing in the sense of the Loewner order. Furthermore, as $\Sigma_{i,j}^{-1}$ is proportional to the SNR, increasing the SNR also enlarges the Loewner order of these matrices being summed and thus leads the partial order of $M_{2}$ to enhance. As a result, the MCRB is a non-increasing function of $N_{T}$, $N_{L}$, or SNR. While an increase of $N_{T}$, $N_{L}$, or SNR improves the robustness of the estimator against noise, these parameters do not have an impact on the feasibility of the estimation. The number of observations required to make proper estimation feasible follows from the analysis from [24] dealing with intrinsic parameter estimation for cameras. Translating the results of that paper to the problem at hand, it follows that observations at two different poses provide sufficient constraints to avoid depth ambiguity, and at each pose, at least four LEDs should be observed to have enough information for the calibration.

In this paper, we estimated the intrinsic parameters from the normalized differences instead of the quad-tuple of RSS values, where the PDF of the normalized differences was approximated by a Gaussian distribution. To evaluate the effect of both the approximation and normalized differencing, we compare the misspecified Cramér-Rao bound $\mathrm{MCRB}\left(\Theta \right)$ (30) with the Cramér-Rao bound for the quad-tuple of RSS values $\overset{˘}{y}$ observed by the QPD, i.e., $\mathrm{CRB}\left(\Theta \right)$, corresponding to the true PDF $p(\overset{˘}{y})$. As $\overset{˘}{y}$ is Gaussian distributed, the derivation of the Fisher information matrix $J\left(\Theta \right)$ and thus $\mathrm{CRB}\left(\Theta \right)=J^{-1}\left(\Theta \right)$ [30] is straightforward and yields

(36) $$J\left(\Theta \right)={\left(\nabla_{\Theta }\overset{˘}{g}\right)}^{T}{\overset{˘}{\Sigma }}_{\eta }^{-1}\left(\nabla_{\Theta }\overset{˘}{g}\right),$$

where $\nabla_{\Theta }\overset{˘}{g}={ [\nabla_{\Theta }g_{1,1},\nabla_{\Theta }g_{1,2},\ldots ,\nabla_{\Theta }g_{N_{T},N_{L}}]}^{T}\in R^{\left(4N_{T}N_{L}\right)\times \left(6N_{T}+3\right)}$ denotes the gradient of $\overset{˘}{g}$ with respect to Θ.

### 3.3. Pose Estimation

Once the intrinsic parameters are estimated during the calibration process, the estimated parameters can be used for accurate pose estimation of the receiver. To estimate the pose, a similar procedure can be used for the calibration by means of the Gauss–Newton method, except that now it is assumed that the intrinsic parameters k are known. At pose *i*, the observation $t_{i}$ only depends on the pose $T_{i}$ and is independent of other poses. Therefore, we can estimate the poses independently, in contrast to the calibration procedure that needed to consider the observations of multiple poses due to the presence of the unknown intrinsic parameters. Again, the estimation is obtained with an iterative procedure of which the update step is the same as in (28):

(37) $$T_{i}^{t+1}=\exp\left({\left(\tau_{T}\Delta_{T_{i}}\left(k,T_{i}^{t}\right)\right)}^{\wedge }\right)T_{i}^{t}$$

Similar to the calibration procedure, we can obtain a coarse estimate by means of the direct linear transformation method together with (29) to initialize the Gauss–Newton method for pose estimation.

To evaluate the pose estimates, the position error is expressed by the Euclidean distance between the position vector r and its estimate $\hat{r}$, i.e., $r_{e}=\hat{r}-r$, while the orientation error is expressed by the axis-angle vector between the orientation matrix R and its estimate $\hat{R}$, i.e., $u_{e}={\left(\log\left(\hat{R}R^{T}\right)\right)}_{⊗}$ [27]. Similar to the pose estimator derived from the calibration algorithm, we can derive the theoretical lower bounds for the pose estimate, i.e., $\mathrm{MCRB}(T_{i})$ and $\mathrm{CRB}(T_{i})$, from (35) and (36), respectively, by restricting the parameters Θ to $T_{i}$. Then the left upper and right bottom 3×3 submatrices of $\mathrm{MCRB}(T_{i})$ are bounds on position and orientation, respectively. The same result applies to $\mathrm{CRB}(T_{i})$.

## 4. Numerical Assessment

In this section, we first verify the performance of the proposed calibration algorithm and compare the mean squared error with the theoretical bound based on simulations. Then, the calibration procedure is used to determine the intrinsic parameters in a simulation setup, after which the pose is estimated. To obtain a comparative study, this section gives a comparison between our proposed pose estimation method and the camera’s PnP and iterative methods.

### 4.1. Calibration

As the number of parameters to be estimated in the calibration phase is large, to have good performance, the number and quality of observations must be sufficiently high. To ensure that these concerns are met, we consider a dedicated calibration setup, where we place the LEDs in a plane at vertical distance sufficiently close to the receiver, i.e., z=0.65 m, to obtain a sufficiently high SNR. Furthermore, to guarantee the receiver has sufficient LEDs within its FOV, we assume $N_{L}=N_{L,r}^{2}$ LEDs are placed in a square grid with an area of 400 cm${}^{2}$, where the LED in the ith row and jth column has position $r_{L}=\left(\frac{20}{N_{L,r}-1} [\begin{array}{c} i-1\\ j-1\\ 0 \end{array}]- [\begin{array}{c} 10\\ 10\\ 0 \end{array}]\right)$ cm, $i,j\in \{1,\ldots ,N_{L,r}\}$. All LEDs are assumed to have a transmitted power $P_{t}=1$ W, and a Lambertian pattern with order γ=1. We set the origin of the system frame as the center of the LED plane.

The receiver consists of a circular QPD with radius $r_{p}=5$ mm, resulting in an active area $A_{p}=\pi r_{p}^{2}$, and responsivity $R_{p}=0.4$ A/W. Above the QPD, we place at a height |h|=3.0 mm a circular aperture with radius l=2.5 mm. In our simulations, we set the misalignment between the centers of the aperture and the QPD equal to $k_{m,q}= [0.5,-0.3]$ mm. The noise in the different quadrants of the QPD is assumed to have a correlation coefficient ρ=0.7. The orientation R of the receiver is defined with the ZXZ Euler angles $\left[\theta_{\alpha },\theta_{\beta },\theta_{\gamma }\right]$ [31]. We assume the azimuth and roll angles, i.e., $\theta_{\alpha }$ and $\theta_{\gamma }$, are uniformly distributed over [0,2π), and we set the elevation angle $\theta_{\beta }$ as a variable. In this way, the receiver has a random orientation but a controlled tilt angle of $\theta_{\beta }$. After fixing the orientation, we still need to specify the position of the receiver. To ensure that most LEDs are observed, we select the position of the receiver so that it is pointing toward the center of the LEDs plane. This corresponds to the position $r=\frac{zRe_{3}}{e_{3}^{T}Re_{3}}$.

In the simulations, we use (15) to generate the observations. We assume the shot noise has power spectral density $N_{0}=2.10\times 10^{-22}A^{2}/\mathrm{Hz}$, which corresponds to a background spectral irradiance $p_{n}=5.8\times 10^{-6}\;W/\left({\mathrm{cm}}^{2}\cdot \mathrm{nm}\right)$ and a visible light bandwidth Δλ=360 nm [32]. Supposing the electrical bandwidth equals B=1 MHz [8], we can compute the noise variance $\sigma_{w3}^{2}$ with $\sigma_{w3}^{2}=N_{0}B$. Simulation results will be given in terms of the effective SNR $={\left(\frac{\left(\gamma +1\right)A_{a}R_{t}P_{t}}{2\pi \sigma_{w3}}\right)}^{2}$ with $A_{a}=\pi l^{2}$.(As the received SNR depends on the receiver pose, a fixed received SNR will limit the parameter space of the receiver pose. As this will complicate the simulation, we chose to fix the effective SNR) Given the optical transmitted power $P_{t}=1$ W, we obtain the effective SNR = 44.73 dB. In our simulations, we will use the range SNR ∈[25,65] dB to take into account variations of the system parameters.

**Figure 5:**
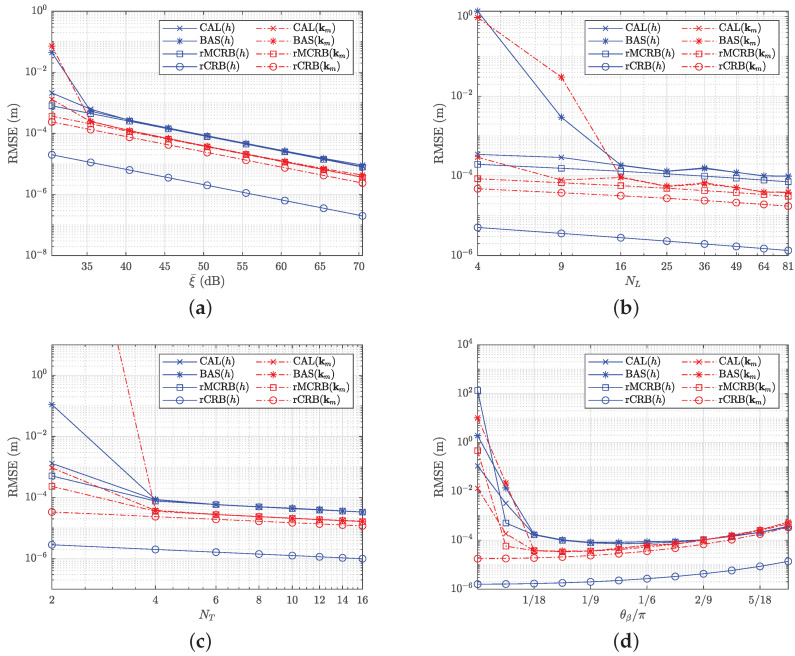
RMSE of the estimator and the theoretical bound: (**a**) $N_{L}=25$, $N_{T}=4$, $\theta_{\beta }=\pi /9$ rad; (**b**) SNR=45 dB, $N_{T}=3$, $\theta_{\beta }=\pi /9$ rad; (**c**) SNR=45 dB, $N_{L}=25$, $\theta_{\beta }=\pi /9$ rad; (**d**) SNR=45 dB, $N_{L}=25$, $N_{T}=4$.

First, we evaluate the performance of the designed calibration estimator as a function of SNR. As a baseline method, we consider the calibration algorithm for cameras [33]. Because the algorithm of [33] takes the image of an object as input, which in this case corresponds to the observation of the light spot’s position, we use (29) prior to this algorithm to convert the observation t to the estimated center of the light spot. The number of LEDs in the LED plane is set to $N_{L}=25$, and the receiver observes the plane from $N_{T}=4$ randomly generated poses with $\theta_{\beta }=\pi /9$ rad. We vary the SNR from 25 to 65 dB and plot in Figure 5a the resulting root mean square error (RMSE) (denoted by CAL) versus $\bar{\xi }$, i.e., the received SNR averaged over the four poses. The root of the MCRB and CRB (denoted by rMCRB and rCRB) from Section 3.2 and the performance of the baseline method (denoted by BAS) are also plotted for comparison. As we can see from Figure 5a, at high $\bar{\xi }$, the two calibration methods perform close to optimal, while at low $\bar{\xi }$, the proposed calibration algorithm outperforms the baseline method. This can be explained as the bias in (29) for the baseline method is larger than for the proposed calibration method when $\bar{\xi }$ is low. The gap between MCRB and CRB reflects the net performance cost of the followed strategy, where we use the normalized differences instead of the RSS values and the approximated PDF. The gap shows that the robustness to imperfect knowledge of the transmitted power and radiation pattern of the LEDs, obtained by this strategy, is achieved at the cost of accuracy. The performance loss for the estimation of *h* is larger than for the estimation of $k_{m}$.

Next, we evaluate the effect of the number $N_{L}$ of LEDs and the number $N_{T}$ of poses. We set SNR =45 dB and $\theta_{\beta }=\pi /9$ rad. Both variables influence the number of observations, so we expect that increasing either $N_{L}$ or $N_{T}$ will improve the performance of the calibration. In Figure 5b, we show the performance for $N_{L,r}=\sqrt{N_{L}}=\left\{2,3,\ldots ,9\right\}$ with $N_{T}=3$, and in Figure 5c for $N_{T}=\left\{2,4,\ldots ,16\right\}$ with $N_{L}=25$. The figures show that the lower bound is reached for both the proposed calibration method and the baseline method when $N_{L}$ or $N_{T}$ is sufficiently large. In that case, the performance improves logarithmically with $N_{L}$ and $N_{T}$. When $N_{L}$ or $N_{T}$ are small, i.e., when the number of observations is small, the proposed method outperforms the baseline method.

Finally, we evaluate the effect of the orientation $\theta_{\beta }$ of the receiver with respect to the LED plane. We set SNR =45 dB, $N_{L}=25$, and $N_{T}=4$. The calibration performance for $\theta_{\beta }$ varying from 0 rad to 5π/18 rad is shown in Figure 5d. When $\theta_{\beta }$ is small, the performance first improves by increasing $\theta_{\beta }$. This can be explained as for small $\theta_{\beta }$, the randomly generated poses are very close to each other implying the solution set is tightly coupled and thus more susceptible to noise. For large $\theta_{\beta }$, the performance starts to degrade, as in our assumptions we assumed that the receiver is pointing to the center of the LEDs plane, implying large $\theta_{\beta }$ corresponds to placing the receiver far from the center of the LEDs plane. As a consequence, the RSS values will experience lower SNR values, causing the slight degradation of the performance. The optimal performance will therefore occur at intermediate values of $\theta_{\beta }$.

### 4.2. Pose Estimation

**Figure 6:**
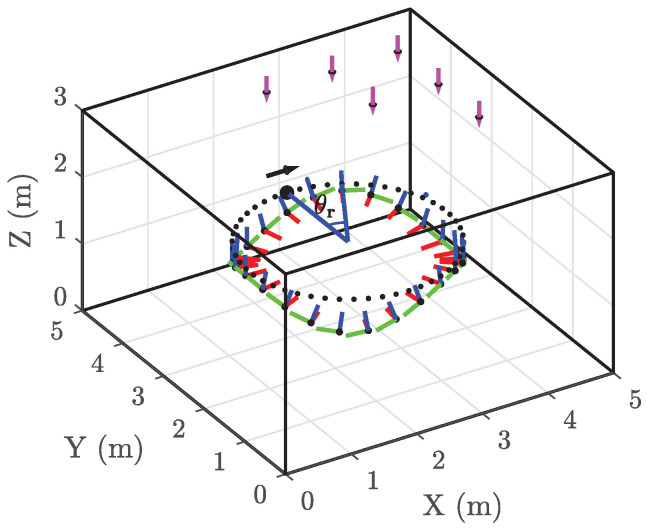
Simulation setup for the QADA receiver. The three orthonormal vectors in three different colors (the red, green, and blue vector represent the *x*-axis, *y*-axis, and *z*-axis, respectively) at each sample on the path represent the frame of the receiver. The pink arrows represent the LEDs (only a fraction of them are shown) on the ceiling. $\theta_{r}$ indicates the traveled angle along the dotted circle in the XY plane.

In the pose estimation phase, we assume the intrinsic parameters are known, i.e., in practice, we use the intrinsic values obtained in the calibration phase. As in this case, we only need to estimate the six parameters of the pose; the number of LEDs can be lower than for the calibration phase. Furthermore, we assume the VLP system is combined with the illumination system, so the number and placement of the LEDs must satisfy the illumination standards. To this end, we consider in the evaluation of the pose estimator a 5m×5m×3m area, where $N_{L}=25$ LEDs with optical power $P_{t}=5$ W are mounted at the ceiling of the area, as shown in Figure 6, unless specified otherwise (assuming the optical power of the LEDs equals 5 W, an average horizontal illuminance of 500 lm (corresponding to an office area) in a plane at z=2 m below the LEDs requires roughly 25 LEDs). We define the boundary vector $b={ [5,5,3]}^{T}$, the number of LED columns in the *X* direction $N_{L,X}=5$ and *Y* direction $N_{L,Y}=5$, and the positions of the LEDs are given by ${ [\frac{{ [b]}_{1}\left(2i-1\right)}{2N_{L,X}},\frac{{ [b]}_{2}\left(2j-1\right)}{2N_{L,Y}},{ [b]}_{3}]}^{T}$, with $i\in \{1,\ldots ,N_{L,X}\}$ and $j\in \{1,\ldots ,N_{L,Y}\}$, where ${\left[b\right]}_{i}$ denotes the ith component of b. We assume the algorithm only uses the LEDs that result in all quadrants to produce a sufficiently large RSS output to ensure that the information in the RSS values is reliable. To take into account the effect of calibration and the possible uncertainty in the estimated parameters, we assume that the receiver has been calibrated and that the estimated intrinsic parameter vector $\hat{k}$∼$N(k,\Sigma_{\hat{k}})$, with $\Sigma_{\hat{k}}=\mathrm{diag}\left(\left[\sigma_{uv}^{2},\sigma_{uv}^{2},\sigma_{h}^{2}\right]\right)$, and $\sigma_{uv}$ and $\sigma_{h}$ are set to the RMSE of the calibration given in the Section 4.1.

To evaluate the performance of the proposed estimators, we consider a path with a circular pattern in the XY plane and a sinusoidal pattern in the *Z* direction as shown in Figure 6. The radius of the circle in the XY plane is 1.5 m, and the amplitude of the sinus is 0.2 m. The circle is centered at ${ [2.5,2.5,1.2]}^{T}$ m. Starting at the coordinates ${ [4.0,2.5,1.2]}^{T}$ m, the path oscillates sinusoidally in the *Z* direction and completes the path in three periods.

**Figure 7:**
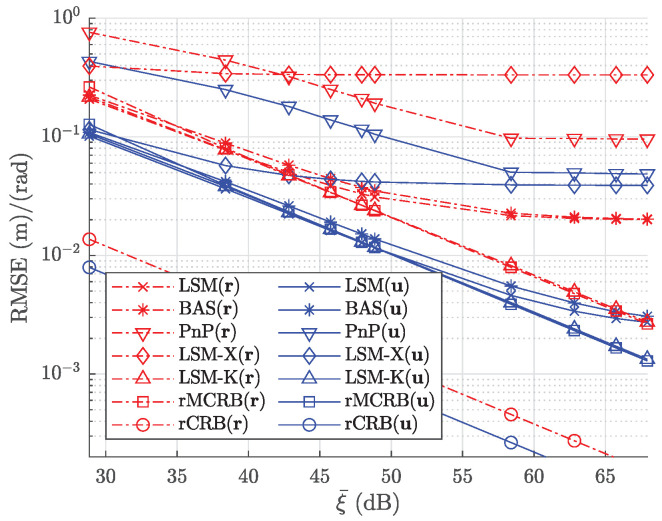
RMSE for position and orientation estimates, compared with the mean received SNR $\bar{\xi }$.

In Figure 7, we evaluate the proposed estimator with respect to the transmitted power. In this simulation, we set $\Sigma_{\hat{k}}$ equal to the corresponding RMSE values from the calibration when SNR = 55 dB, i.e., $\sigma_{uv}=1.17\times 10^{-5}$ m and $\sigma_{h}=2.60\times 10^{-5}$ m. The RMSE of the pose estimate, averaged over the path, is first evaluated in terms of the transmitted power $P_{t}=\left(1+2m\right)\times 10^{n}$ W, where the variables m∈{0,…,4} and n∈{0,1} specify the selected power values in the figure. Figure 7 shows this averaged RMSE of the designed estimator based on the least squares method (LSM) (denoted by LSM) and the root of the MCRB (denoted by rMCRB) as a function of the mean received SNR $\bar{\xi }$. In comparison, we also add the performance of the proposed estimator when perfect knowledge of the intrinsic parameters is given (denoted by LSM-K) and when the intrinsic parameters are not calibrated and assumed to be the design values |h|=2.8 mm and $k_{m,q}=0$ mm (denoted by LSM-X), while the true values are randomly selected from a Gaussian distribution with as averages the design values and variances given above. The averaged RMSE of the baseline method [33] (denoted by BAS) and the root of the CRB (denoted by rCRB) are also plotted for comparison. As a second baseline method, we also consider the classical PnP method (denoted by PnP). As expected, the performance of the estimator with perfect knowledge of the intrinsic parameters reaches the theoretical lower bound MRCB and improves with the SNR. On the other hand, the performance of the estimators without perfect knowledge of the intrinsic parameters, i.e., calibrated or not, shows an error floor when the received SNR increases. This error floor is caused by the uncertainty in the estimated intrinsic parameters, which becomes dominant when the received SNR is sufficiently large. Obviously, the error floor is much higher in the absence of calibration. Our algorithm strongly outperforms classical PnP, as expected, while the its performance is slightly better than the baseline method BAS. The gap between MCRB and CRB implies that the robustness to imperfect knowledge of the transmitted power and the radiation pattern of the LEDs, obtained by the strategy of normalizing differences, is achieved at the cost of accuracy.

**Figure 8:**
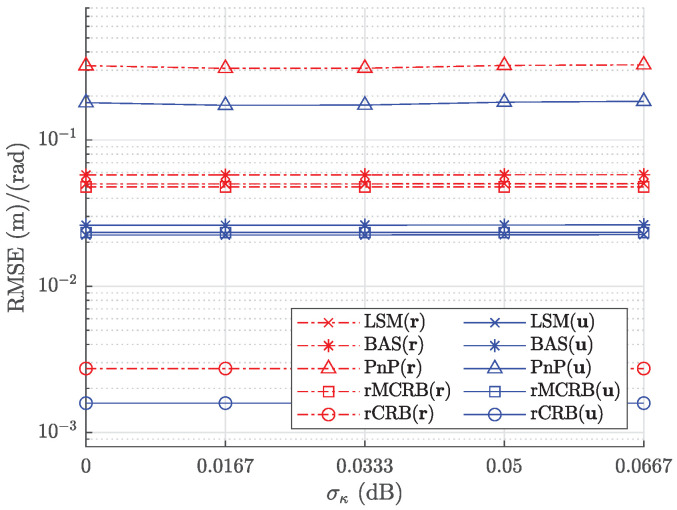
RMSE for position and orientation estimates as a function of $\sigma_{\kappa }$.

To illustrate the robustness of the proposed algorithm to fluctuations in the transmitted power, we consider the situation where the transmitted power of an LED follows a Gaussian distribution [34], i.e., $P_{t}=\kappa P_{t,m}$, with $\kappa ∼N(1,\sigma_{\kappa }^{2})$. Experiments in [34] show that the standard deviation $\sigma_{\kappa }$ in general is smaller than $\sigma_{\kappa }<0.0667$. In Figure 8, we plot the RMSE averaged over the entire path as a function of the standard deviation $\sigma_{\kappa }\in \left[0,0.0667\right]$, assuming $P_{t,m}=5$ W, $\sigma_{uv}=1.17\times 10^{-5}$ m, and $\sigma_{h}=2.60\times 10^{-5}$ m. We observe that for the given range of $\sigma_{\kappa }$, the RMSE is (essentially) independent of $\sigma_{\kappa }$, implying the approach of the normalized differences is robust to fluctuations in the transmitted power.

**Figure 9:**
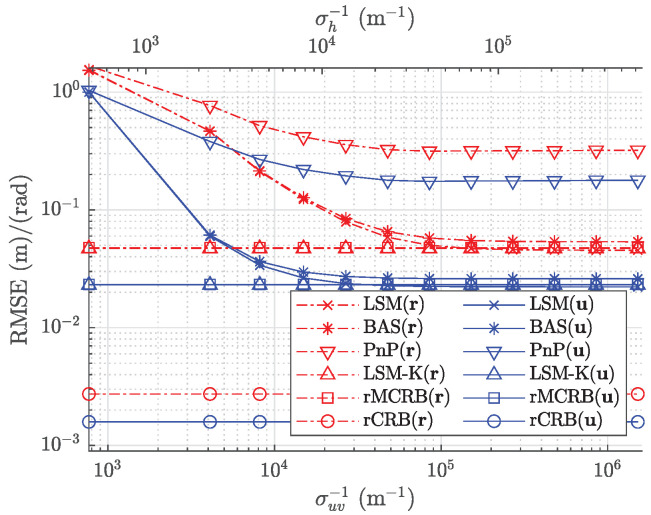
RMSE for position and orientation estimates as a function of $\sigma_{uv}$ and $\sigma_{h}$.

Next, we show the RMSE of the pose estimates of the proposed estimator as a function of the uncertainty in $\hat{k}$ for $P_{t}=5$ W and compare the resulting RMSE with that of the baseline method and with the theoretical lower bounds. Figure 9 shows that, as expected, the performance of the estimator with the perfect knowledge of the intrinsic parameters reaches the theoretical lower bound and is independent of the uncertainty. The figure also illustrates that a large uncertainty degrades the performance of the estimators significantly. When the uncertainty becomes smaller, the performance of the estimators of LSM and BAS gradually reach and are bounded by the theoretical lower bound. This is expected, as due to the uncertainty, the estimators are in the best case as good as the ones with perfect knowledge of the intrinsic parameters. (Close to) optimal performance is achieved as long as the uncertainty is sufficiently small.

**Figure 10:**
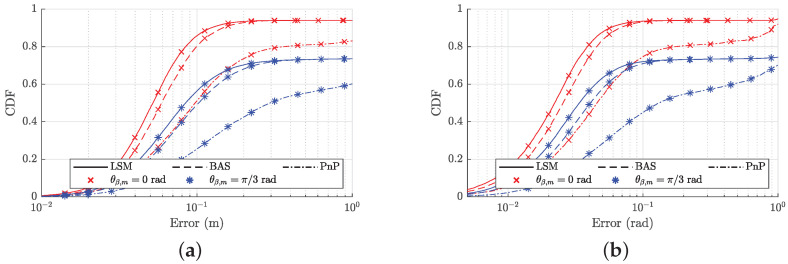
CDF of RMSE for position and orientation estimates for $z_{d}=2$ m: (**a**) position error; (**b**) orientation error.

**Figure 11:**
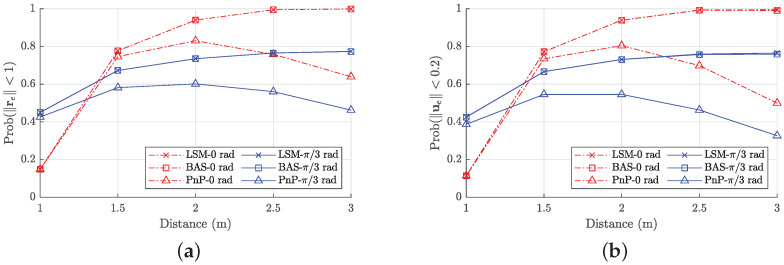
Probability versus the distance $z_{d}$ for: (**a**) position error $∥r_{e}∥<1$ m; (**b**) orientation error $∥u_{e}∥<0.2$ rad.

Finally, we evaluate the performance of the proposed receiver over the whole considered area. For the LEDs, we consider the setup of Figure 6, where the $N_{L}=25$ LEDs are attached in a square grid to the ceiling of the 5 m × 5 m area. We assume the receiver is placed at the positions belonging to a horizontal grid with a horizontal spacing of 0.2 m, at a vertical distance $z_{d}\in \left\{1,1.5,2,2.5,3\right\}$ m from the LEDs. Taking into account that the receiver in practice is pointing roughly upwards, we consider for each sample point a random orientation with an elevation angle $\theta_{\beta }$ that is uniformly distributed within the interval $[0,\theta_{\beta ,m}]$ rad. We consider two scenarios, i.e., $\theta_{\beta ,m}=0$ rad for the case where the receiver is always pointing upwards, and $\theta_{\beta ,m}=\pi /3$ rad for the tilted scenario. Figure 10 shows the cumulative distribution (CDF) of the pose error when $z_{d}=2$ m. We observe that the proposed algorithm outperforms the baseline methods BAS and PnP. The figure further reveals that our algorithm converges approximately for 95% of the sample positions when $\theta_{\beta ,m}=0$ rad, i.e., when the receiver points upwards, while this reduces to 75% when a random tilted angle is considered, i.e., when $\theta_{\beta ,m}=\pi /3$ rad. Hence, as expected, the coverage degrades if the receiver has a random tilt. To evaluate the effect of the vertical distance $z_{d}$ on the performance, we compute the probability that the error is smaller than 1 m for the position and 0.2 rad for the orientation. Figure 11 shows that the performance of our estimator improves when the distance increases, as well as for the baseline method BAS. Despite the larger distance, and thus lower SNR, increasing the distance will bring more LEDs into the field-of-view of the receiver, implying more information is available to estimate the pose. On the other hand, for the PnP method, the performance degrades when the distance increases. This is explained as the PnP method is more sensitive to a reduction of the SNR, resulting in a performance degradation despite the larger number of LEDs available within the field-of-view.

## 5. Conclusions

In this paper, we investigate the pose estimation and calibration problem for the QADA receiver. To this end, the channel link is first modeled in terms of the receiver’s pose and misalignment using the perspective projection model. Then, we study the effect of the strategy of differencing and normalization to the probability density function (PDF), and it is shown that the applied strategy results in a complex PDF. Therefore, we derive an approximated PDF in a simple closed-form, based on which the calibration and the pose estimation algorithms using the least squares principle are proposed. The proposed calibration does not require any information about the pose of the receiver and is robust to the received power variation and imperfect knowledge of the LEDs’ radiation patterns, while the proposed pose estimator is able to directly estimate the pose from the (normalized) RSS values. Computer simulations confirm that the proposed calibration algorithm is effective and that the proposed pose estimator outperforms the PnP algorithm.

## Data Availability

The study did not report any data.

## Appendix A. The Expressions for the Gradient ∇_Θ*μi,j*_

Based on the chain rule, $\nabla_{\Theta }\mu_{i,j}=\nabla_{r_{S,q,i,j}}\mu_{i,j}\nabla_{\Theta }r_{S,q,i,j}\in R^{2\times \left(6N_{T}+3\right)}$, where $\nabla_{r_{S,q,i,j}}\mu_{i,j}$ is the gradient of $\mu_{i,j}$ with respect to $r_{S,q,i,j}$, and $\nabla_{\Theta }r_{S,q,i,j}$ is the gradient of $r_{S,q,i,j}$ with respect to Θ. $\nabla_{r_{S,q,i,j}}\mu_{i,j}$ is determined by its $k^{th}$ component

(A1) $${ [\nabla_{r_{S,q,i,j}}\mu_{i,j}]}_{k}=\frac{\left(2-2\cos2\alpha_{k}\right)e_{k}^{T}}{l\pi \sqrt{1-{\left(e_{k}^{T}r_{S,q,i,j}/l\right)}^{2}}},$$

while $\nabla_{\Theta }r_{S,q,i,j}= [\nabla_{k}r_{S,q,i,j},\nabla_{T_{1}}r_{S,q,i,j},\ldots ,\nabla_{T_{N_{T}}}r_{S,q,i,j}]\in R^{2\times \left(6N_{T}+3\right)}$ is given by

(A2) $$\nabla_{k}r_{S,q,i,j}=\frac{{\left(T_{i}{\bar{r}}_{L,j}\right)}^{©}}{e_{3}^{T}T_{i}{\bar{r}}_{L,j}}$$

and

(A3) $$\nabla_{T_{k}}r_{S,q,i,j}=\left\{\begin{array}{ccc} \left(\frac{K}{e_{3}^{T}T_{i}{\bar{r}}_{L,j}}-\frac{KT_{i}{\bar{r}}_{L,j}e_{3}^{T}}{{\left(e_{3}^{T}T_{i}{\bar{r}}_{L,j}\right)}^{2}}\right){\left(T_{i}{\bar{r}}_{L,j}\right)}^{⊙} & \; & \mathrm{if}\;k=i\\ 0\in R^{2\times 6} & \; & \mathrm{if}\;k\neq i \end{array}\right.,$$

where the gradient of $r_{S,q,i,j}$ with respect to $T_{k}$ (A3) is calculated with the infinitesimal perturbation, and the operators $\cdot^{©}$ and $\cdot^{⊙}$ are defined as

(A4) $${\left({ [a_{1},a_{2},a_{3},a_{4}]}^{T}\right)}^{©}= [\begin{array}{ccc} a_{3} & 0 & a_{1}\\ 0 & a_{3} & a_{2} \end{array}],$$

and

(A5) $${\left({ [\xi^{T},\eta ]}^{T}\right)}^{⊙}= [\begin{array}{cc} \eta I_{3} & -\xi_{\times }\\ 0^{T} & 0^{T} \end{array}],$$

respectively.
